# An Interventional Study Exploring the Effects of Nutritional Psychoeducation on Emotional Eating After 3 Months

**DOI:** 10.3390/medsci13030138

**Published:** 2025-08-16

**Authors:** Maria Mentzelou, Gavriela Voulgaridou, Konstantinos Papadimitriou, Olga Alexatou, Eirini-Georgia Deligiannidou, Aspasia Serdari, Sousana K. Papadopoulou, Evmorfia Psara, Gerasimos Tsourouflis, Constantinos Giaginis

**Affiliations:** 1 Department of Food Science and Nutrition, School of the Environment, University of the Aegean, 81400 Myrina, Greece; maria.mentzelou@hotmail.com (M.M.); fnsd23003@fns.aegean.gr (O.A.); fnsd21013@fns.aegean.gr (E.P.); 2 Department of Physiotherapy, School of Health Sciences, International Hellenic University, 57001 Thessaloniki, Greece; gabivoulg@gmail.com; 3 Department of Nutritional Sciences and Dietetics, School of Health Sciences, International Hellenic University, 57400 Thessaloniki, Greece; kpapadimitriou@ihu.gr (K.P.); deligiannidoueirini@yahoo.gr (E.-G.D.); souzpapa@gmail.com (S.K.P.); 4 Department of Medicine, School of Health Sciences, Democritus University of Thrace, 68100 Alexandroupolis, Greece; 5 Department of Psychiatry, School of Medicine, Democritus University of Thrace, 68100 Alexandroupolis, Greece; aserdari@yahoo.com; 6 Second Department of Propedeutic Surgery, Medical School, University of Athens, 11527 Athens, Greece; gtsourouflis@med.uoa.gr

**Keywords:** emotional eating, clinical trial, motivation, nutritional psychoeducation, intervention, BMI

## Abstract

**Background/Objectives**: Emotional eating may be a consequence of acquired cue reactivity, a lack of control, or an inaccurate link between episodic overeating and negative affect, according to a new analysis of its standard measurement. This study was a controlled trial, which was designed to investigate the effect of personalized nutritional psychoeducation on emotional eating behavior. **Methods:** This study enrolled 95 participants (62 control group and 33 intervention group) who were randomized to treatment and assessed at 3-month follow-up. Over a period of 3 months, six thematic individual sessions were conducted with a frequency of every 15 days for the participants in the intervention group. The Three-Factor Eating Questionnaire (TFEQ) was used to assess feeding patterns and the manifestation of emotional food consumption in response to emotion. **Results:** At baseline, it appears that gender is positively related to TFEQ Emotional Score (β: 1.77 (random error: 0.57, *p*-value: 0.003). BMI (β: −0.11 (random error: 0.04, *p*-value: 0.003) and waist circumference are negatively related to the TFEQ Emotional Score. **Conclusions:** Although this study confirmed significant associations of emotional eating and gender, BMI, and waist circumference, the nutritional psychoeducation-based intervention did not exert the expected effects on emotional eating. More high-quality clinical trials need to further be designed to improve emotional eating behavior by applying relevant nutritional psychoeducation-based interventions.

## 1. Introduction

Being hungry is only one aspect of eating, which is a basic necessity. Numerous factors, including heredity, hormones, religious views, emotion, media, and environment, influence eating behavior. It has been demonstrated that there is a significant correlation between eating behavior and food intake [1]. Another essential component of human existence, activity, and functioning is emotion, which is a response to the cognitive evaluation of environmental stimuli. Emotions are tightly linked to various emotional and health effects, as well as to food choices and the social ritual of feeding [2,3]. The tendency to overeat in response to negative emotions, such as anxiety or irritability, is known as emotional eating [4]. Researchers now see it as a reaction to both happy and negative emotions. In eating disorders, emotional eating may be a symptom of broader issues with controlling emotions. Emotional eating is also associated with a number of physical and mental health issues, such as mood swings, depression, binge eating, and fluctuations in body weight [5,6].

The idea of emotional eating is more complicated than it first appears, and a new analysis of its usual measurement suggests that it may be a reflection of acquired cue reactivity, a lack of control, or an incorrect association between episodic overeating and bad affect. In this regard, the present measurement of emotional eating may be a reflection of the “worry” or “overconcern” that accompanies disinhibited eating, which is fueled by decreased inhibitory control and acquired hedonic cue responsiveness, particularly during periods of high emotion [7,8]. Moreover, the affect regulation model states that eating is a coping strategy used by those who feel more negative emotions in an effort to lessen them [9]. However, negative emotions continue following sessions of emotional eating, demonstrating that emotional eating is ineffective at controlling emotions [10,11].

Emotional eating may be unproductive since it serves to avoid rather than accept the feeling of negative emotions, which may be linked to the guilt that comes with overeating. Interventions that assist emotional eaters in better coping with unpleasant emotions and reducing emotional eating may be beneficial, as emotional eating is ineffectual in controlling negative emotions [12]. In addition, emotional eating behavior appears to be governed in part by classical conditioning of appetitive reaction to actual or predicted negative emotions. Correlations of the unidimensional measures of emotional eating may reveal additional aspects of the emotional eating construct. Reduced inhibitory control, a diminished capacity to distinguish internal body signals, stress perception, intuitive eating, and emotional regulation issues are a few of these [13,14].

As a symptom of emotion dysregulation linked to mental and physical health issues, emotional eating must also be addressed from the standpoint of symptom reduction. Weight issues, including weight increase over time and trouble losing weight, have frequently been researched in relation to emotional eating [15]. However, there is growing evidence that bad eating habits are a stronger indicator of physical health than weight or BMI, and emotional eating leads to an unhealthy diet by boosting the consumption of high-calorie items [10,16].

According to the surveys conducted, there were very few studies about the role of emotional eating in nutritional intake. Identifying emotional and non-emotional eating patterns, Mediterranean Diet adherence, anxiety and emotional eating, and sleep quality in female health science students during the final wave of the COVID-19 pandemic was the aim of Diaz-Urena’s et al. cross-sectional study. It was detected that emotional eaters have worse adherence to the Mediterranean Diet than non-emotional eaters and they often prefer energy-dense foods with abundant saturated fat [17]. A representative sample of King Abdulaziz University college students participated in this cross-sectional study of Shatwan et al. to examine the relationship between persistent stress and emotional eating and adherence to the Healthy Eating Index (HEI) with non-emotional eaters having higher HEI scores than other emotional groups. Also, there was a positive association with unhealthy eating habits [18].

In order to ascertain if anxiety and emotional eating were associated with food habits, the cross-sectional study analyzed the dietary, alcohol, emotional eating, and anxiety patterns of a sample of women from a Spanish university. Fast food and sweets or less healthy eating habits were strongly linked to emotional eating among children [19]. Zare’s et al. study examined the relationship between emotional eating and nutritional intake in Iranian women with overweight and obesity. They found that high amounts of hyper-tasty energy-dense foods that contain high fat and sugar levels could be a reason for higher energy intake in emotional eaters [20]. The purpose of Ljubičić’s study was to find correlations between food intake and emotional eating behaviors that result from emotional states such as stress, depression, loneliness, the urge to stay awake, relaxation, and self-satisfaction. According to the findings, people who were more emotionally motivated and have conditioned eating habits are more likely to eat when they are stressed, depressed, lonely, or bored [21].

Based on our literature review, there is no other kind of nutritional intervention among Greek adults that examined emotional eating. The aim of this intervention was to evaluate the effects of personalized nutritional psychoeducation on eating behaviors, particularly cognitive restraint, uncontrolled eating, and emotional eating. A basic component of the intervention is mindfulness training in order to help individuals to connect with hunger cues and satiety and manage cravings. Personalized nutrition treatments provide tailored nutritional advice, goods, or services based on individual characteristics. Personalization can improve the effectiveness of standardized nutrition interventions by addressing individual variability. Personalized nutrition interventions involve adapting information to each individual depending on feedback, ensuring a better match. Additionally, the study sought to assess whether the intervention’s impact on psychological factors, including depression, anxiety, and stress, influence eating behavior. Furthermore, we examined the extent to which adherence to the Mediterranean Diet (MedDiet) was associated with changes in eating behavior.

## 2. Methods

### 2.1. Study Design and Sample

The present study is a controlled trial examining the effect of personalized nutritional psychoeducation on emotional eating behavior, psychological state, and adherence to the Mediterranean Diet (MedDiet). Participants were recruited at random through community advertisements and screened for eligibility criteria, including age between 18 and 75 years and absence of chronic illnesses (eating disorders, metabolic disorders, cardiovascular diseases, cancer, or mental illnesses). All participants provided written informed consent after being informed of the study objectives and data confidentiality procedures. Participants were assigned alternately to the intervention and control groups in a 2:1 ratio based on the order of recruitment (e.g., first participant to the control group, second to the intervention group, third to the control group, and so on). While this allocation method ensured balanced group sizes, it did not involve allocation concealment.

Sample size was calculated using a repeated measures ANOVA design. Based on a medium effect size (f = 0.25), α = 0.05, power (1-β) = 0.80, two measurement points, and correlation among repeated measures = 0.5, the required sample size was 80 participants. Accounting for an anticipated 20% dropout rate, we aimed to recruit 96 participants. Participants were randomly allocated to the intervention (*n* = 64) or control (*n* = 32) groups using a 2:1 ratio to increase the precision of treatment effect estimates, as recommended for intervention studies with resource constraints.

The survey was performed in agreement with the Declaration of Helsinki and approved by the Institutional Ethics Committee of the University of the Aegean (protocol code 1218/12.4.2022 and date of approval: 12 April 2022). The participant recruitment for the intervention began in January 2023. The baseline assessments began at the end of August 2024 and the intervention began in the middle of October 2024.

### 2.2. Sociodemographic and Anthropometric Parameter Evaluation

First, their sociodemographic information (gender, age, education level, marital status, and smoking status) was collected in the beginning of the study. Anthropometric measurements were made at the beginning of the trial to determine body weight and whether obesity was present. The same precise testing equipment and measuring technique were employed for this purpose. Because the devices utilized were portable, taking measurements was made simple. In order to further evaluate overweight or obesity, qualified professional dietitians used the following measurements: height, body weight, body mass index (BMI), hip circumference (HC), waist circumference (WC), and waist-to-hip ratio (WHR) [22].

### 2.3. Questionnaires

To conduct the study, the completion of all participant questionnaires was carried out in face-to-face interviews in a calm environment to respect the privacy of the process.

The Three-Factor Eating Questionnaire (18-item), a weighted Greek version questionnaire, was used to assess feeding patterns and the manifestation of emotional food consumption in response to emotion in the Greek adult population. It also assesses several forms of restrictive and uncontrolled eating behavior at the same time [23]. Cronbach’s alpha, an index of TFEQ reliability, was estimated to be 0.81.

The Depression Anxiety Stress Scale (21-item) is suitable for general use in both clinical and non-clinical adult populations that assesses symptoms of depression, anxiety, and stress in the community. It is important to emphasize that the Greek translation of the DASS has been shown to be a valid and reliable tool [24].

By giving consumption of the MedDiet’s products a score between 0 and 5, where 5 denotes the maximum adherence, Panagiotakos et al. developed and explained MedDiet score adherence. The total score ranges from zero to fifty-five points. The researchers assigned scores 0 for those who reported no consumption and 1 to 5 for those who reported rare to daily consumption of foods thought to be similar to this pattern (i.e., those recommended daily or more than three portions per week, i.e., non-refined cereals, fruits, vegetables, legumes, olive oil, fish, and potatoes) [25].

Measurements were conducted before the intervention (baseline, study week 00) and after the 12-week intensive intervention period (post-intervention, study week 10).

### 2.4. Intervention

Over a period of 3 months, 6 thematic individual 1 h sessions, which were based on motivational interviewing (MI) and mindful-based eating awareness training (MB-EAT), were conducted with a frequency of every 15 days for the participants in the intervention group. The pillars of the nutritional psychoeducation intervention involved discussions on the following issues: stress and anxiety management for body image, nutritional management of difficult situations during periods of weight management, physical activity, education on natural hunger and satiety cues, nutritional management of meals outside the home, and improving knowledge about food. A summary of the course curriculum is provided in Table 1.

### 2.5. Statistical Analysis

Continuous variables were summarized using mean and standard deviation (SD) for normally distributed data or median and interquartile range (IQR) for non-normally distributed data. Categorical variables were expressed as frequencies and percentages. The normality of distribution of continuous variables was assessed using the Shapiro–Wilk test and via visual inspection of histograms and Q-Q plots. At both time points, weight, BMI, age, waist circumference, waist-to-hip ratio, emotional eating (TFEQ), and DASS score consistently showed non-normal distributions. Hip circumference and TFEQ cognitive restraint approached normality (*p* > 0.05) at several time points, while the Mediterranean Diet Score exhibited mixed results, with normality only at baseline but not at 3 months. When examined by group, similar patterns were observed: the control group showed non-normal distributions for most variables, whereas the intervention group exhibited normality only for hip circumference and TFEQ cognitive restraint. 

Initial comparisons between groups (control vs. intervention) were performed using either the Welch’s *t*-test or the Mann–Whitney test and between time points for each group, e.g., t0 control vs. t2 control, paired *t*-test, or Wilcoxon signed-rank test.

Linear mixed-effects models were used to analyze changes in emotional eating over time, accounting for within-subject correlation. We tested five hierarchical models with increasing complexity to determine whether the model, including the time × group interaction, provided a better fit compared to models with only time, only group, or the additive effects of time and group.

- Model 1 (Null): Y_ij_ = β_0_ + u_i_ + ε_ij_
- Model 2 (Time): Y_ij_ = β_0_ + β_1_Time_ij_ + u_i_ + ε_ij_
- Model 3 (Group): Y_ij_ = β_0_ + β_1_Group_i_ + u_i_ + ε_ij_
- Model 4 (Additive): Y_ij_ = β_0_ + β_1_Time_ij_ + β_2_Group_i_ + u_i_ + ε_ij_
- Model 5 (Interaction): Y_ij_ = β_0_ + β_1_Time_ij_ + β_2_Groupi + β_3_ (Time × Group)_ij_ + u_i_ + ε_ij_

Where Y_ij_ represents the outcome for participant i at time j, u_i_ represents random intercept for participant i, and ε_ij_ represents residual error. Model comparison was performed using Akaike Information Criterion (AIC), Bayesian Information Criterion (BIC), and likelihood ratio tests. The compound symmetry covariance structure was specified based on theoretical considerations and empirical fit. For estimation of the degrees of freedom in the linear mixed-effects models, we applied the Kenward–Roger approximation. Assumptions regarding covariance structure were assessed, and a compound symmetry structure was adopted based on model fit. Standardized regression coefficients (β standardized) were calculated to assess effect sizes for continuous predictors. The interpretation of β standardized interpretation was as follows: 0.1 = small effect, 0.3 = medium effect, 0.5 = large effect. For the primary interaction effect, we calculated the proportion of variance explained. Confidence intervals (95% CI) were computed for all parameter estimates using the profile likelihood method. Stepwise model selection using AIC and BIC was employed to identify significant predictors of emotional eating, including demographic (age, gender, education), anthropometric (BMI, waist circumference), and psychological variables (DASS subscales).

All analyses were conducted in R version 4.3.1 using the pwr package for sample size calculation, the psych package for descriptive statistics, the lme4 package for mixed-effects models, the lmerTest package for significance testing, and the effectsize package for standardized coefficient estimation. SPSS version 29.0.2.0 was used only for preliminary descriptive statistics. The analysis code is available upon request.

## 3. Results

We included 62 participants for the control group and 33 for the intervention group. All participants completed the follow-up and there were not any dropouts. Table 2 and Table 3 present the characteristics for both groups, control and intervention, for baseline (t0) and 3 months (t1). At baseline (t0), no significant differences were observed between the intervention and control groups across demographic, anthropometric, or psychological variables, confirming successful randomization, while the proportion of women was higher in both groups (control: 77.4%, intervention: 69.7%). At baseline (t0), participants in the intervention group had significantly higher weight (median 82.8 kg vs. 67.0 kg, *p* = 0.001), BMI (28.5 vs. 24.6 kg/m^2^, *p* = 0.001), and hip circumference (111.5 vs. 101.3 cm, *p* = 0.001) compared to the control group. The proportion of participants with overweight or obesity was also higher in the intervention group (75.8% vs. 46.8%, *p* = 0.012). Additionally, marital status differed significantly between groups, with a greater proportion of married participants in the intervention group (84.8% vs. 62.9%, *p* = 0.046). Waist circumference was higher in the intervention group (*p* = 0.017), although the waist-to-hip ratio did not differ significantly. Among psychological measures, TFEQ uncontrolled eating scores were slightly lower in the intervention group at baseline (*p* = 0.019), while other TFEQ subscales, DASS scores, and Mediterranean Diet scores showed no significant baseline differences. At 3 months (t1), there were no significant differences between groups in weight, BMI, or the majority of psychological and dietary measures, suggesting that the intervention did not produce clear between-group differences within this timeframe. Depression, anxiety, and stress categories as well as Mediterranean Diet adherence quartiles also remained comparable between groups. Overall, baseline imbalances were observed mainly in anthropometric parameters, whereas post-intervention differences were not statistically significant (Table 2).

Between time points t0 and t1, the number of participants in the two groups remained stable. Regarding the psychological parameters, the values of the TFEQ (Uncontrolled, Cognitive Restraint, Emotional) and the total score remained similar at the two time points. Correspondingly, the DASS scores for anxiety, depression, and stress did not show significant changes, while the proportion of participants classified in “Normal” or “Moderate” categories for psychological distress remained consistent throughout the intervention period. Finally, the score on the Med Diet and the distribution of participants in its quartiles remained stable. Overall, no significant changes were identified between the two time points, indicating that the two groups maintained similar characteristics during the intervention (Table 3).

Table 4 presents model comparison statistics for the five linear mixed-effects models tested. The time × group interaction was not statistically significant (β = −0.49, SE = 0.42, 95% CI [−1.31, 0.33], *p* = 0.243, indicating that changes in emotional eating over the 3-month period did not differ significantly between the intervention and control groups. The inclusion of standardized coefficients (β) allows for comparison of the relative effect sizes across predictors within and between models. Across all models, the standardized coefficients for time (β ranging from −0.02 to 0.04) and the group effect (β ≈ −0.25) were small, and their 95% confidence intervals included zero, indicating that these effects were not statistically significant. Similarly, the time × group interaction in Model M5 showed a small, standardized effect (β = −0.18, 95% CI [−0.48, 0.13]) and did not reach significance. Although Model M3 had the lowest AIC value, suggesting slightly better fit, Model M5 was selected for further interpretation because it incorporates the interaction term, aligning with the study’s hypothesis that changes in emotional eating may differ between groups over time.

Mixed-effects models were conducted to examine the associations of demographic, anthropometric, psychological, and dietary factors with emotional eating scores (Table 5). Male gender was positively associated with higher emotional eating (b = 1.77, *p* = 0.003, β = 0.67), while both BMI and waist circumference were inversely associated with emotional eating (BMI: b = −0.11, *p* = 0.003, β = −0.29; waist circumference: b = −0.04, *p* = 0.013, β = −0.20). Neither time, group assignment, nor their interaction showed significant effects on emotional eating. Among psychological variables, moderate and extremely severe depression showed trends toward lower emotional eating scores, though they did not reach significance. Anxiety and stress categories, as well as Mediterranean Diet Score and quartiles, were not significantly associated with emotional eating.

In Table 6, the results of the best multivariate mixed model for the estimated β coefficients (random error) of mixed linear models of the TFEQ emotional relationship are presented. This model was derived using the stepwise method based on the values of the statistical criteria AIC and BIC (835.18, 876.61).

The results of the best multivariate mixed model for TFEQ emotional show that regarding gender (gender), men exhibited a mean value of β = 2.47 (SE= 0.58), with a *p*-value < 0.001, indicating that gender has a statistically significant effect on the dependent variable TFEQ emotional. Specifically, this difference is very significant, as the *p*-value is less than 0.05, indicating that the effect of gender is significant for explaining the variability in the score, regardless of time and group.

Regarding educational level, the values for the various education categories (university, Master’s, Doctorate) did not show a statistically significant effect. The beta values were negative, but with *p*-values greater than 0.05 for all categories (university: −1.22 with *p*-value = 0.144: Master’s: −1.29 with *p*-value = 0.143: Doctorate: −0.50 with *p*-value = 0.661), indicating that education is not significantly related to TFEQ Emotional.

In Table 6, the final multivariate mixed-effects model for emotional eating includes several predictors and accounts for individual variability with random intercepts. Gender had a significant positive effect (β = 0.91), indicating that men tend to have higher emotional eating scores compared to women, with a relatively large effect size. Waist circumference showed a significant negative standardized effect (β = −0.38), suggesting that participants with larger waist circumference reported lower emotional eating scores, which is a moderate effect. Educational level variables were not statistically significant, with negative standardized coefficients suggesting a trend toward lower emotional eating scores among those with higher education, but confidence intervals crossing zero indicate uncertainty in these effects. Regarding time and group effects, the standardized coefficient for time (3 months vs. baseline) was small and non-significant (β = 0.04), indicating little overall change in emotional eating scores across the study period. The intervention group effect was also negligible and non-significant (β = −0.03), meaning no baseline difference between groups. The interaction term between time and the intervention group, which tests whether the intervention altered the change in emotional eating over time compared to the controls, was negative but non-significant (β = −0.18). This suggests that while the intervention group showed a slight reduction in emotional eating scores over time relative to the controls, this difference was not statistically meaningful. In summary, the standardized coefficients highlight that gender and waist circumference are the most influential predictors in this model, whereas the intervention did not produce a significant effect on emotional eating changes over the 3-month period. The stepwise selection process ensured that these predictors represent the best fitting model based on the data.

## 4. Discussion

This study investigated the effects of nutritional intervention that were delivered in the intervention group among adults through nutritional psychoeducation and motivational interviewing. MI is an evidence-based, time-limited, person-centered therapy method to increase a person’s motivation and commitment to behavior change. There was no evidence of an effect on emotional eating at intervention end. The main findings of the present study are summarized as follows: The effects of gender and waist circumference are important to explain the variability of the score, irrespective of time and group. The intervention also improved psychological factors known to contribute to long-term success in weight management. It was noted that this trend was also true when controlling for depression and stress. Data on emotional eating in Greece are scarce. The originality of the study is that it is the first intervention that has taken place in a healthy adult Greek population following our literature review.

Bibliographic comparisons show that interventions are in principle mainly directed at patients with overweight and obesity. However, when based on nutritional advice alone, they often do not result in statistically significant reductions in emotional eating. On the contrary, significant benefits are observed when nutritional support is combined with behavioral modification techniques.

In terms of the association between emotional score and waist circumference, EE scale scores frequently correspond with binge eating; however, the findings are inconsistent. It is not universally true that EE has a direct effect on body weight and waist circumference. Emotional feeding is also seen in populations with a healthy BMI. Previous research has shown the impact of attitudes toward emotional expression on EE and body weight, helping to support current advances in an affect phobia model of EE. It should also be noted that mindful awareness skills’ potential for prevention may be restricted. Another study indicated that the susceptibility to EE did not change between normal-weight adults and adults with obesity, suggesting that EE should be a worry for normal-weight people as well [26].

Based on the gender findings in the literature, EE may be more common among women. This disparity could be attributed to women’s social pressures and expectations around thinness and body image, which are frequently promoted on social media. Women may be more dissatisfied with their bodies and utilize exercise as a coping mechanism to deal with stress and negative emotions. This type of consumption has been associated with anxiety and sadness in the general population, as well as certain gender-specific distinctions. EE has been strongly associated with anxiety symptoms in women and depression symptoms in males [27].

Our findings were consistent with those reported by Barnes et al.; the study tested intervention that was 3-months in length with 12-month follow-up assessment. Researchers compared motivational interviewing and nutritional psychoeducation, and they did not find any statistically significant difference for weight loss [28]. Similarly, the study of Moreira et al. evaluated the effect of a counseling approach based on mindful eating on the eating behavior of individuals living with overweight and obesity. The results did not show statistically significant improvements in emotional eating behavior based on individual online nutritional counseling through a 16-week randomized clinical trial [29].

Furthermore, Kearney et al.’s mindfulness-based stress reduction intervention with participants (*N* = 48) was not associated with significant changes in EE. In order to determine whether this publicly accessible program has an impact on eating, the study’s goal was to investigate the connection between mindfulness-based stress reduction (MBSR) participation and eating patterns. The current study found no evidence of a decrease in EE linked to MBSR involvement, nor did it uncover any notable changes in the consumption of various dietary categories, such as total energy, fat, sugar, fruit, and vegetables [30]. Furthermore, Kudlek et al. in their comprehensive systematic review and meta-analysis of RCTs, found that behavioral weight management interventions have small to moderate improvements for numerous eating behavior traits at intervention end [31]. The main factor may account for the lack of change in measures of EE. Notably, we found evidence of reductions in emotional eating scores when we controlled for depressive and stress symptoms. At the conclusion of the intervention and the 12-month follow-up, they did not find any evidence of an effect on emotional eating. Due to the limited number of trials reporting data, they were unable to make trustworthy findings for a range of eating behavioral traits. These outcomes included internal regulation, pleasure, reward, contextual skills, hedonic hunger, disinhibition, external eating, susceptibility to hunger, intuitive/mindful eating, and contextual skills. Because of the small number of contributing trials, significant heterogeneity, large prediction intervals, and potential for publication bias, results from all outcomes should be considered cautiously [31].

In contrast, Schnepper et al. investigated the effectiveness of a four-session mindfulness and prolonged chewing intervention and showed a decrease in emotional eating after 8 weeks. The dependent variables were body mass index and food craving, as well as emotional, external, and intuitive eating [32]. Moreover, women with obesity participating in a community-based obesity treatment centered around self-regulatory skills to control eating were evaluated over 6 months and exhibited reduced emotional eating [33].

A pilot study has recently made a valuable contribution to the scant literature by indicating the feasibility, acceptability, and preliminary effectiveness of combining somatic, multisensory, and cognitive manipulations delivered via telemedicine to help patients with emotional eating to manage their emotions [34]. This pilot study included 21 participants who received seven weekly one-hour virtual experiences focusing on emotional regulation. A greater improvement in emotional eating in the virtual experience emotional regulation group and a significant reduction in emotion dysregulation after the treatment were observed compared to the typical treatment [34].

Another randomized controlled trial intervention was performed on 148 boys and girls in primary school [35]. In this study, the interventional group engaged in fitness yoga, kickboxing, and/or spinning sessions, and mindfulness practices twice per week for 12 weeks. On the other hand, the control group engaged in a recreational play session once per week for 12 weeks. The interventional group showed lower scores in emotional eating, anxiety, and sleep latency post-intervention compared to the control group, highlighting that mindfulness-based intervention may be promising for the management of emotional eating [35].

A cable-TV-delivered weight control randomized trial, including 331 Black women (aged 18–75 years; BMI ≥ 25 kg/m^2^), was performed at baseline and 3, 8, and 12 months post-randomization [34]. At 3 months (immediately postintervention), BMI decreased for women in all “eating when depressed or sad” (EWD) and “eating to manage stress” (ETMS) categories but enhanced at later follow-up for women reporting EWD and ETMS always/often. In addition, 8-month EWD and ETMS predicted 12-month BMI. Higher perceived stress was associated with higher EWD and ETMS; however, stress was not associated with lagged BMI or waist circumference at any time. This study supported evidence that addressing emotional eating and related triggers could improve weight maintenance in interventions with Black women [36].

A controlled pragmatic trial was also performed to explore the effects of Pythagorean Self Awareness Intervention (PSAI), a cognitive stress management program in a primary school setting [37]. Participants were randomly assigned to either the intervention group (*n* = 23) or the control group (*n* = 22). At the end of the 8-week period, the participants in the intervention group experienced significant reductions in stress, anxiety, guilt, and emotional eating and an increase in MedDiet and pride [36]. Thus, this study supported evidence that PSAI had positive effects on primary school attendants’ emotional eating and psychological state [37].

The limitations of the current study should be taken into account. First, there was no feedback on anthropometric parameters at the end of the intervention. Second, there was no usual care comparator condition for this time point of the long-term outcome. Moreover, research shows that people underestimate the effect of emotions on their behavior when they are not emotionally active. Another drawback is the possibility that memory bias or social desirability may affect responses, especially in the evaluation of emotional eating. It should be noted that the majority of the sample is female, which introduces a bias. Despite the limitations of the study, the finding of a strong correlation between changes in nutrition–psychological education and changes in EE merit further investigation in future studies. The results of this study reinforce the view that emotional eating is a multi-factorial phenomenon, influenced by biological, psychological, and nutritional parameters.

## 5. Conclusions

This is one of the first studies to assess the impact of an intervention strategy to reduce emotional eating in adults. The influence of gender and waist circumference on emotional eating is a key factor. In addition, the contribution of depression and stress is a finding that requires further investigation. However, future research is needed to explore how to better promote the development of additional psychological support for weight management programs that focus on emotional eating. Studies with longer intervention periods should also be conducted.

## Figures and Tables

**Table 1:** List of weekly topics for intervention group.

Weeks	Topic
**0**	Recognizing the connections between ideas, emotions, and actions Negative thought patterns Recognizing and breaking harmful thought patterns
**2**	Advice for effective, long-term weight control Review every week Dealing with weight plateaus
**4**	Exercise Changing activities associated with eating Staying motivated
**6**	Hunger that is internal as opposed to exterior (and emotional) Taking care of internal hunger by adhering to a meal plan Mindful eating of a pleasurable or “risky” food
**8**	Eating out Individuals as catalysts for poor decision-making Acting contrary to your feelings
**10**	Food pyramid and portion control Monitoring calories Goal setting and behavior change

**Table 2:** Characteristics of study participants at baseline (t0 = 0 months) and 3 months (t1 = 3 months) (control vs. intervention).

Variable	Level	t0 Control (*n* = 62)	t0 Intervention (*n* = 33)	*p*	t1 Control (*n* = 62)	t1 Intervention (*n* = 33)	*p*
**Gender**	Female	48 (77.4%)	23 (69.7%)	0.564	–	–	–
	Male	14 (22.6%)	10 (30.3%)		–	–	–
**Age (years)**		35.0 [29.2, 40.8]	31.0 [28.0, 38.0]	0.316	–	–	–
**Employment Status**	Unemployed	14 (22.6%)	4 (12.1%)	0.335	–	–	–
	Employed	48 (77.4%)	29 (87.9%)		–	–	–
**Marital Status**	Single	23 (37.1%)	5 (15.2%)	0.046	–	–	–
	Married	39 (62.9%)	28 (84.8%)		–	–	–
**Education Level**	High school	8 (12.9%)	2 (6.1%)	0.653	–	–	–
	University	27 (43.5%)	17 (51.5%)		–	–	–
	MSc	21 (33.9%)	12 (36.4%)		–	–	–
	PhD	6 (9.7%)	2 (6.1%)		–	–	–
**Smoking**	Non-smoker	53 (85.5%)	25 (75.8%)	0.370	–	–	–
	Smoker	9 (14.5%)	8 (24.2%)		–	–	–
**Height (cm)**		1.7 [1.6, 1.7]	1.7 [1.6, 1.8]	0.407	–	–	–
**Weight (kg)**		67.0 [60.0, 82.8]	82.8 [73.0, 92.7]	0.001	–	–	–
**BMI (kg/m^2^)**		24.6 [21.5, 27.7]	28.5 [25.3, 34.1]	0.001	–	–	–
**BMI Categories**	Underweight	2 (3.2%)	1 (3.0%)	0.049	–	–	–
	Normal	31 (50.0%)	7 (21.2%)		–	–	–
	Overweight	18 (29.0%)	14 (42.4%)		–	–	–
	Obese	11 (17.7%)	11 (33.3%)		–	–	–
	Overweight/Obese	29 (46.8%)	25 (75.8%)		–	–	–
**Waist Circumference (cm)**		83.0 [72.0, 92.0]	90.0 [82.0, 105.0]	0.017	–	–	–
**Waist Circumference Assessment**	Normal	15 (24.2%)	14 (42.4%)	0.109	–	–	–
	Increased	47 (75.8%)	19 (57.6%)		–	–	–
**Waist-to-Hip Ratio**		0.8 (0.1)	0.8 (0.1)	0.558	–	–	–
**WHR Assessment**	Normal	10 (16.4%)	3 (9.1%)	0.505	–	–	–
	Increased	51 (83.6%)	30 (90.9%)		–	–	–
**TFEQ Uncontrolled**		24.9 (3.0)	23.2 (3.6)	0.019	24.8 (3.4)	24.0 (4.1)	0.279
**TFEQ Cognitive Restraint**		15.1 (2.4)	15.7 (2.2)	0.230	15.8 (2.3)	15.4 (2.4)	0.467
**TFEQ Emotional**		8.5 [6.0, 10.8]	8.0 [6.0, 10.0]	0.496	9.0 [6.2, 10.8]	8.0 [6.0, 10.0]	0.114
**TFEQ Total Score**		48.3 (5.2)	46.9 (5.9)	0.214	49.1 (5.6)	46.9 (6.4)	0.099
**DASS**		7.0 [3.0, 15.0]	10.0 [3.0, 18.0]	0.438	6.0 [3.0, 13.0]	6.0 [0.0, 16.0]	0.944
**Depression**	Mild	6 (9.7%)	4 (12.1%)	0.168	10 (16.1%)	2 (6.1%)	0.439
	Normal	37 (59.7%)	15 (45.5%)		38 (61.3%)	20 (60.6%)	
	Moderate	8 (12.9%)	8 (24.2%)		9 (14.5%)	5 (15.2%)	
	Severe	5 (8.1%)	0 (0.0%)		2 (3.2%)	3 (9.1%)	
	Extremely Severe	6 (9.7%)	6 (18.2%)		3 (4.8%)	3 (9.1%)	
**Anxiety**	Mild	4 (6.5%)	2 (6.1%)	0.555	5 (8.1%)	1 (3.0%)	0.334
	Normal	33 (53.2%)	13 (39.4%)		33 (53.2%)	19 (57.6%)	
	Moderate	7 (11.3%)	5 (15.2%)		11 (17.7%)	2 (6.1%)	
	Severe	5 (8.1%)	6 (18.2%)		7 (11.3%)	5 (15.2%)	
	Extremely Severe	13 (21.0%)	7 (21.2%)		6 (9.7%)	6 (18.2%)	
**Stress**	Mild	6 (9.7%)	6 (18.2%)	0.113	5 (8.1%)	5 (15.2%)	0.231
	Normal	44 (71.0%)	20 (60.6%)		49 (79.0%)	22 (66.7%)	
	Moderate	5 (8.1%)	1 (3.0%)		5 (8.1%)	1 (3.0%)	
	Severe	2 (3.2%)	5 (15.2%)		1 (1.6%)	3 (9.1%)	
	Extremely Severe	5 (8.1%)	1 (3.0%)		2 (3.2%)	2 (6.1%)	
**Mediterranean Diet Score**		33.0 [31.0, 36.0]	33.0 [32.0, 37.0]	0.580	34.0 [30.0, 37.0]	34.0 [32.0, 37.0]	0.544
**Mediterranean Diet Quartiles**	1st	15 (24.2%)	7 (21.2%)	0.913	19 (30.6%)	4 (12.1%)	0.101
	2nd	19 (30.6%)	11 (33.3%)		11 (17.7%)	9 (27.3%)	
	3rd	14 (22.6%)	6 (18.2%)		13 (21.0%)	12 (36.4%)	
	4th	14 (22.6%)	9 (27.3%)		19 (30.6%)	8 (24.2%)	

Continuous variables presented as mean (standard deviation) or median (Q1–Q3), categorical variables as percentage (frequency). Abbreviations: BMI = body mass index; TFEQ = Three-Factor Eating Questionnaire; DASS-21 = Depression Anxiety Stress Scales-21 item version; score ranges: TFEQ Emotional (3–12), Mediterranean Diet Score (0–14), DASS-21 subscales (0–42 each; anthropometric data were measured only at baseline (t0); *p*-values correspond to comparisons between groups using Welch’s *t*-test/Mann–Whitney test or Chi-square tests, as appropriate).

**Table 3:** Characteristics of study participants by treatment group at baseline (t0) vs. 3 months (t1).

		t_1_ =0 VS. t_2_ = 3 months			t_1_ =0 VS. t_2_ = 3 months		
Variable	Level	Control	Control	*p*-Value	Intervention	Intervention	*p*-Value
***N***		62	62		33	33	
**Gender**	Women	48 (77.4)	48 (77.4)		23 (69.7)	23 (69.7)	
	Men	14 (22.6)	14 (22.6)		10 (30.3)	10 (30.3)	
**TFEQ Uncontrolled**		24.9 (3.0)	24.8 (3.4)	0.933	23.2 (3.6)	24.0 (4.1)	0.428
**TFEQ Cognitive Restraint**		15.1 (2.4)	15.8 (2.3)	0.127	15.7 (2.2)	15.4 (2.4)	0.564
**TFEQ Emotional**		8.5 [6.0, 10.8]	9.0 [6.2, 10.8]	0.764	8.0 [6.0, 10.0]	8.0 [6.0, 10.0]	0.547
**TFEQ Τotal Score**		48.3 (5.2)	49.1 (5.6)	0.457	46.9 (5.9)	46.9 (6.4)	0.968
**DASS**		7.0 [3.0, 15.0]	6.0 [3.0, 13.0]	0.556	10.0 [3.0, 18.0]	6.0 [0.0, 16.0]	0.318
**Depression**	Mild	6 (9.7)	10 (16.1)	0.500	4 (12.1)	2 (6.1)	0.194
	Normal	37 (59.7)	38 (61.3)		15 (45.5)	20 (60.6)	
	Moderate	8 (12.9)	9 (14.5)		8 (24.2)	5 (15.2)	
	Severe	5 (8.1)	2 (3.2)		0 (0.0)	3 (9.1)	
	Extremely Severe	6 (9.7)	3 (4.8)		6 (18.2)	3 (9.1)	
Anxiety	Mild	4 (6.5)	5 (8.1)	0.418	2 (6.1)	1 (3.0)	0.573
	Normal	33 (53.2)	33 (53.2)		13 (39.4)	19 (57.6)	
	Moderate	7 (11.3)	11 (17.7)		5 (15.2)	2 (6.1)	
	Severe	5 (8.1)	7 (11.3)		6 (18.2)	5 (15.2)	
	Extremely Severe	13 (21.0)	6 (9.7)		7 (21.2)	6 (18.2)	
Stress	Mild	6 (9.7)	5 (8.1)	0.740	6 (18.2)	5 (15.2)	0.907
	Normal	44 (71.0)	49 (79.0)		20 (60.6)	22 (66.7)	
	Moderate	5 (8.1)	5 (8.1)		1 (3.0)	1 (3.0)	
	Severe	2 (3.2)	1 (1.6)		5 (15.2)	3 (9.1)	
	Extremely Severe	5 (8.1)	2 (3.2)		1 (3.0)	2 (6.1)	
Med Diet		33.0 [31.0, 36.0]	34.0 [30.0, 37.0]	0.606	33.0 [32.0, 37.0]	34.0 [32.0, 37.0]	0.524
Med Diet quartiles	1ο	15 (24.2)	19 (30.6)	0.334	7 (21.2)	4 (12.1)	0.380
	2ο	19 (30.6)	11 (17.7)		11 (33.3)	9 (27.3)	
	3ο	14 (22.6)	13 (21.0)		6 (18.2)	12 (36.4)	
	4ο	14 (22.6)	19 (30.6)		9 (27.3)	8 (24.2)	

Continuous variables presented as mean (standard deviation) or median (Q1–Q3); categorical variables as percentage (frequency). Abbreviations: TFEQ = Three-Factor Eating Questionnaire; DASS-21 = Depression Anxiety Stress Scales-21 item version; score ranges: TFEQ Emotional (3–12), Mediterranean Diet Score (0–14), DASS-21 subscales (0–42 each); *p*-values correspond to comparisons between groups using paired *t*-test/Wilcoxon signed-rank test or Chi-square tests, as appropriate.

**Table 4:** Comparative presentation of mixed-effects models for TFEQ Emotional Eating (unstandardized and standardized coefficients).

Parameter	Time Model (M2)	Group Model (M3)	Time + Group Model (M4)	Interaction Model (M5)
	**b (SE, *p*)**	**b (SE, *p*)**	**b (SE, *p*)**	**b (SE, *p*)**
	**β [95% CI]**	**β [95% CI]**	**β [95% CI]**	**β [95% CI]**
**Intercept (baseline control)**	8.21 (0.28), *p* < 0.001	8.42 (0.32), *p* < 0.001	8.45 (0.34), *p* < 0.001	8.35 (0.35), *p* < 0.001
	0.001 [−0.24, 0.25]	0.09 [−0.15, 0.32]	0.10 [−0.15, 0.34]	0.06 [−0.19, 0.32]
**Time (t1 = 3 months vs. t0 = baseline)**	−0.04 (0.20), *p* = 0.834	–	−0.04 (0.20), *p* = 0.834	−0.13 (0.25), *p* = 0.603
	0.02 [−0.05, 0.09]	–	−0.02 [−0.17, 0.13]	0.04 [−0.14, 0.23]
**Group (intervention vs. control)**	–	−0.68 (0.54), *p* = 0.214	−0.68 (0.54), *p* = 0.214	−0.42 (0.59), *p* = 0.482
	–	−0.25 [−0.64, 0.15]	−0.25 [−0.64, 0.15]	−0.15 [−0.59, 0.28]
**Time × Group interaction**	–	–	–	−0.49 (0.42), *p* = 0.243
	–	–	–	−0.18 [−0.48, 0.13]
**AIC**	852.60	851.09	853.05	855.22
**BIC**	872.08	870.57	875.78	881.02

b = unstandardized regression coefficient; SE = standard error; β = standardized regression coefficient; CI = confidence interval. Models include random intercepts and slopes for participants. Outcome variable: TFEQ Emotional Eating subscale (range 3–12, higher scores indicate greater emotional eating). AIC = Akaike Information Criterion; BIC = Bayesian Information Criterion.

**Table 5:** Mixed-effects models examining predictors of emotional eating (TFEQ Emotional Subscale).

Predictor	b (SE)	*p*-Value	β [95% CI]
**Gender (men vs. women)**	**1.77 (0.57)**	**0.003**	**0.67** [0.24, 1.10]
Time (1 vs. 0)	0.12 (0.25)	0.603	0.04 [−0.14, 0.23]
Intervention group (vs. control)	−0.55 (0.57)	0.339	−0.22 [−0.64, 0.21]
Time × group	−0.49 (0.42)	0.243	−0.18 [−0.48, 0.13]
**BMI (kg/m** **^2^)**	**−0.11 (0.04)**	**0.003**	**−0.29** [−0.48, −0.10]
**Waist circumference (cm)**	**−0.04 (0.02)**	**0.013**	**−0.20 [−0.40, −0.02]**
**DASS Depression (ref: mild/normal)**			
Moderate	−1.01 (0.59)	0.077	−0.39 [−0.82, 0.04]
Severe	0.06 (0.76)	0.934	0.03 [−0.54, 0.60]
Extremely severe	−1.30 (0.68)	0.060	−0.48 [−0.98, 0.02]
**DASS Anxiety (ref: mild/normal)**			
Severe	−0.88 (0.76)	0.251	−0.33 [−0.89, 0.24]
Extremely severe	−0.54 (0.68)	0.426	−0.21 [−0.71, 0.30]
**DASS Stress (ref: mild/normal)**			
Normal	0.84 (0.54)	0.122	0.31 [−0.08, 0.71]
Moderate	0.85 (0.68)	0.214	0.33 [−0.19, 0.84]
Severe	−0.65 (0.69)	0.349	−0.27 [−0.80, 0.26]
**Med Diet Score**	0.04 (0.03)	0.223	0.07 [−0.05, 0.20]
**Med Diet Quartiles (ref: Q1)**			
Q2	0.26 (0.43)	0.543	0.03 [−0.30, 0.35]
Q3	0.06 (0.47)	0.907	0.002 [−0.35, 0.35]
Q4	0.70 (0.49)	0.156	0.20 [−0.16, 0.57]

b = unstandardized regression coefficient; SE = standard error; β = standardized regression coefficient; CI = confidence interval. Each predictor was tested in a separate mixed-effects model adjusting for time and group and their interaction. Random intercepts for participants were included in all models. Bold values indicate statistically significant effects (*p* < 0.05).

**Table 6:** Final multivariate mixed-effects model for emotional eating: stepwise selection results.

Parameter	b (SE)	*p*-Value	Standardized β	95% CI (Standardized β)
**Intercept**	**14.00 (1.75)**	**<0.001**	**0.18**	**[−0.37, 0.74]**
**Gender (men)**	**2.47 (0.58)**	**<0.001**	**0.91**	**[0.48, 1.33]**
**Educational level**				
University	−1.22 (0.83)	0.144	−0.45	[−1.05, 0.15]
Master’s degree	−1.29 (0.87)	0.143	−0.48	[−1.11, 0.16]
PhD	−0.50 (1.13)	0.661	−0.18	[−1.01, 0.64]
**Waist circumference (cm)**	**−0.06 (0.02)**	**<0.001**	**−0.38**	**[−0.58,−0.19]**
**Time (3 months vs** **. baseline)**	0.11 (0.25)	0.648	0.04	[−0.14, 0.23]
**Intervention group**	−0.07 (0.55)	0.897	−0.03	[−0.43, 0.37]
**Time × intervention group**	−0.48 (0.42)	0.261	−0.18	[−0.48, 0.13]

b = unstandardized regression coefficient; SE = standard error; β = standardized regression coefficient; CI = confidence interval. Random intercepts for participants were included in all models. Bold values indicate statistically significant effects (*p* < 0.05).

## Data Availability

Data is available upon request to the corresponding author.
